# Identification of Hub Genes and Construction of a Transcriptional Regulatory Network Associated With Tumor Recurrence in Colorectal Cancer by Weighted Gene Co-expression Network Analysis

**DOI:** 10.3389/fgene.2021.649752

**Published:** 2021-04-07

**Authors:** Shengwei Liu, Fanping Zeng, Guangwen Fan, Qiyong Dong

**Affiliations:** ^1^Department of Pharmacy, Yongchuan Hospital of Chongqing Medical University, Chongqing, China; ^2^Chongqing Key Laboratory of Biochemistry and Molecular Pharmacology, School of Pharmacy, Chongqing Medical University, Chongqing, China

**Keywords:** colorectal cancer, tumor recurrence, hub genes, transcription factors, weighted gene co-expression network analysis

## Abstract

Tumor recurrence is one of the most important risk factors that can negatively affect the survival rate of colorectal cancer (CRC) patients. However, the key regulators dictating this process and their exact mechanisms are understudied. This study aimed to construct a gene co-expression network to predict the hub genes affecting CRC recurrence and to inspect the regulatory network of hub genes and transcription factors (TFs). A total of 177 cases from the GSE17536 dataset were analyzed via weighted gene co-expression network analysis to explore the modules related to CRC recurrence. Functional annotation of the key module genes was assessed through Gene Ontology and Kyoto Encyclopedia of Genes and Genomes analyses. The protein and protein interaction network was then built to screen hub genes. Samples from the Cancer Genome Atlas (TCGA) were further used to validate the hub genes. Construction of a TFs-miRNAs–hub genes network was also conducted using StarBase and Cytoscape approaches. After identification and validation, a total of five genes (TIMP1, SPARCL1, MYL9, TPM2, and CNN1) were selected as hub genes. A regulatory network of TFs-miRNAs-targets with 29 TFs, 58 miRNAs, and five hub genes was instituted, including model GATA6-MIR106A-CNN1, SP4-MIR424-TPM2, SP4-MIR326-MYL9, ETS1-MIR22-TIMP1, and ETS1-MIR22-SPARCL1. In conclusion, the identification of these hub genes and the prediction of the Regulatory relationship of TFs-miRNAs-hub genes may provide a novel insight for understanding the underlying mechanism for CRC recurrence.

## Introduction

Colorectal cancer (CRC) ranks as the third most common type of tumor around the world and accounts for more than 7% of overall cancer-related death in China (Liu et al., 2015; Siegel et al., 2020). Despite the massive progress in understanding its genetic mechanism and improvement of surgical techniques, the overall survival time of CRC patients is still not remarkably improved, which could be ascribed primarily to the high recurrence rates (Yu et al., 2017; Ha et al., 2019; Mirgayazova et al., 2019). Therefore, identifying the key regulators involved in CRC recurrence and deciphering their underlying mechanisms are critical for CRC prognosis and the development of novel therapeutic drugs.

With the recent developments in bioinformatics, a number of well-designed and effective methods are now available for the comprehensive identification of biomarkers in cancer and the prediction of cancer-related signaling pathways (Carter et al., 2004; Carey et al., 2005; Liu et al., 2007). Among them, the weighted gene co-expression network analysis (WGCNA) approach provides a systematic biology strategy to identify modules of highly correlated genes and construct a co-expression gene network (Langfelder and Horvath, 2008). By utilizing the WGCNA algorithm, genes with similar co-expression patterns are classified into a set of modules, in which the most central genes could be further identified as hub genes. Compared with the other methodologies applied to analyze the high-throughput sequencing data, WGCNA implements methods for both weighted and unweighted correlation networks and provides a more effective mean to explore the potential association between modules and sample traits.

In recent years, WGCNA has been widely used to uncover the potential biomarkers associated with clinical parameters in various cancer types (Liang et al., 2020; Wang et al., 2020). In terms of CRC, PIGU was identified as a key modulator that is closely related to KRAS mutant CRC patients (Zhang M. et al., 2020), while FBXW4 was reported to be associated with chemotherapy resistance and prognosis of CRC (Zhang Y. et al., 2020). With the help of WGCNA, a set of four long non-coding RNAs (lncRNAs) were found to be significantly correlated with the carcinogenesis and progression of colon adenocarcinoma (COAD) (Jiang et al., 2019). In addition, some efforts have also been taken to identify the crucial regulators associated with the tumor recurrence of CRC patients through WGCNA (Qiu et al., 2020; Wu et al., 2020). However, the number of hub genes identified in the existed studies is relatively low, and the important upstream mediators of these hub genes remain to be fully investigated.

In the current study, WGCNA was constructed based on the GSE17536 dataset containing gene expression profiling results from 177 CRC patients. The specific module associated with the recurrence status of CRC was identified. More importantly, hub genes that play essential roles during CRC recurrence were dug out, and their upstream miRNAs and transcription factors (TFs) were also explored.

## Materials and Methods

### Data Collection and Pre-processing

The mRNA expression profiling dataset GSE17536 of human CRC with patient clinical information was downloaded from Gene Expression Omnibus (GEO) online database (Smith et al., 2010). GSE17536 dataset was performed on platform GPL570 Affymetrix Human Genome U133 Plus 2.0 Array (HG-U133_Plus_2). GSE17536 was used to construct a co-expression network then distinguishing hub genes, which included 177 CRC samples. R packages were used to annotate the original data, generate an expression matrix, and match probes to target gene symbols. Median absolute deviations (MADs) were arranged from large to small, and the expression of the top 25% genes with the greatest differences in samples was selected for WGCNA.

### Construction of WGCNA

R package “WGCNA” was conducted (Langfelder and Horvath, 2008). Firstly, a similarity matrix was constructed by calculating the correlation of all gene pairs. Secondly, using the pickSoftThreshold function in R language, a suitable soft thresholding power was determined, and the parameters that provide appropriate soft threshold power (β) for network construction were acquired (Botía et al., 2017). After choosing the appropriate β = 4, subsequently, the adjacency of the gene network was transformed into a topological overlap matrix (TOM) followed by a calculation of corresponding dissimilarity (1-TOM). Subsequently, a hierarchical clustering method is used to classify genes with similar expression profiles into the same modules, and, by default, the minimum number of genes for the genes dendrogram is set to 30. To further analyze the module, the dissimilarity of module eigengenes (ME) was calculated the dynamicTreeCut algorithm of WGCNA, and some highly similar modules with the height of ME in the clustering lower than 0.25 were merged. Finally, the characteristic gene network was visualized.

### Identification of Modules Associated With Clinical Features

In this study, two methods were used to identify key modules associated with CRC recurrence. ME are the first principal component of a given module and could be considered as a representative of gene expression profile in a module. Module membership (MM) represents the correlation between genes and ME. Gene significance (GS) refers to the correlation between the genes and clinical data, and the average GS for all the genes in a module was defined as module significance (MS), and the module with the highest absolute MS value was regarded as the module with the most significant association with clinical information. Finally, the dissimilarity of the ME was calculated using the moduleEigengenes function in the R WGCNA package. GS was calculated by linear regression between gene expression and CRC recurrence, and MS related to clinical CRC recurrence was obtained.

### Functional Enrichment Analysis of the Key Module Genes

Gene Ontology enrichment analysis has become a widely used method in functional gene annotation. To further investigate the function of differentially expressed genes (DEGs) in the key module, clusterProfiler R package (Yu et al., 2012)^1^ with a strict cut-off of FDR < 0.05 was used to present GO and Kyoto Encyclopedia of Genes and Genomes (KEGG) pathway analysis. Then, a “ggplot2” R package was used to perform the first 10 enrichment terms of GO analysis and KEGG pathway analysis. The cutoff criterion of *P* < 0.05 was considered to be statistically significant.

### Protein–Protein Interaction of the Key Module Genes

Search Tool for the Retrieval of Interacting Genes (Szklarczyk et al., 2019) (STRING, Version: 11.0^2^) was employed to identify protein–protein interactions (PPIs) with a medium confidence interaction score of 0.4 (the turquoise module). In addition, Cytoscape software (Shannon et al., 2003) was applied to visualize the PPI networks^3^. The Molecular Complex Detection (MCODE) plug-in of Cytoscape tool was used to visualize the significant gene modules in CRC, as default, with degree cut-off = 2, node score cut-off = 0.2, k-core = 2, and max.depth = 100. Furthermore, the criteria for selecting the top four significant modules were set as follows: MCODE scores ≥8 and number of nodes ≥10. In addition, STRING was used to present the co-expression analysis of hub genes.

### Identification and Validation of Hub Genes

Based on the MCODE analysis, the genes of the top clusters were selected as candidate genes for further analysis. The GEPIA (Tang et al., 2017)^4^ is a webserver for analyzing gene expression profiles of 9736 tumors and 8587 normal samples from the Cancer Genome Atlas (TCGA) and the genotype-tissue expression (GTEx) projects. In this study, the Kaplan–Meier plotter was used to plot survival analyses of the top module genes, then the GEPIA webserver was used to confirm outcomes of survival analyses. For each gene, cancer patients were divided into two groups according to the median values of mRNA expression. Moreover, TCGA data of CRC were used to validate the mRNA expression of identified hub genes comparing with normal colon tissues. Therefore, we can verify the transcriptional levels of hub genes in CRC tissues. *P* < 0.01 was considered to be statistically significant.

The Human Protein Atlas^5^ was applied to validate the hub genes by immunohistochemistry (IHC). The cBio Cancer Genomics Portal^6^ version: 2.2.0) is an open-access tool that provides analysis, visualization, and downloading of cancer genomics data sets for multiple tumor types. By using the cBioPortal tool, complex cancer genome profiles can be accessed, and this enables us to compare the genetic variations of the selected hub genes in CRC.

### Construction of TFs-miRNAs–Hub Genes Network

StarBase (Li et al., 2014)^7^, a database for exploring microRNA-mRNA interaction maps, was used to predict miRNAs that bind to hub genes based on the screening criteria that CLIP Data ≥1 and expression was present in at least one tumor sample. Then, miRNAs with most intersections in seven databases (PITA, RNA22, miRmap, microT, miRanda, PicTar, and TargetScan) were selected. A co-expression network on account of correlation analysis of hub genes and cancer-related miRNAs was constructed by Cytoscape software. Then, the plugin iRegulon of Cytoscape is applied to forecast TF regulation networks.

## Results

### Data Pre-processing and Quality Assessment

**FIGURE 1:**
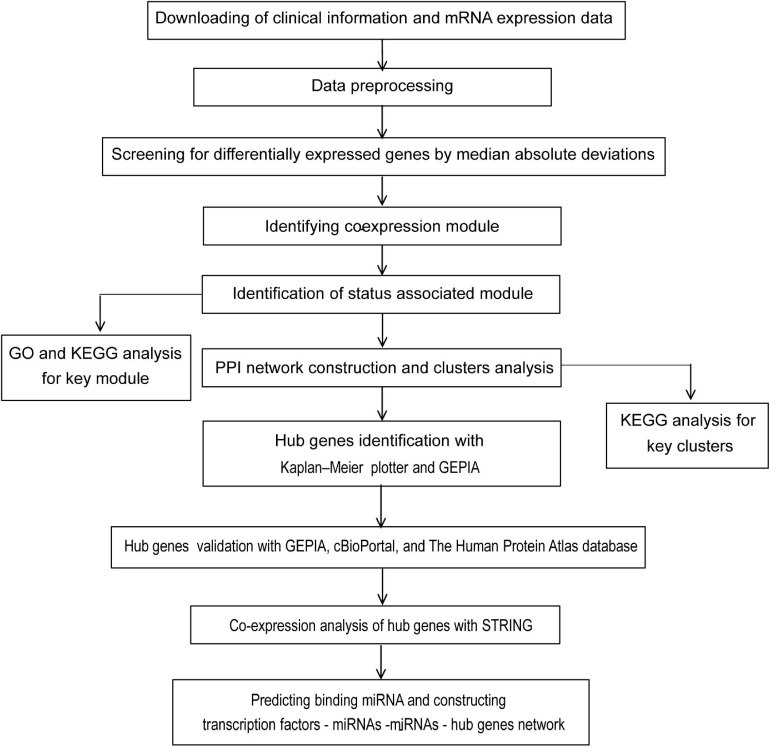
Flow chart of strategy for data preparation, processing, analysis, and validation used in this study.

The flow diagram of the study design is depicted in Figure 1. In general, a total of 177 samples in the GSE17536 dataset were downloaded from the GEO database. After the primary quality control by the WGCNA R package, one outliner sample (Supplementary Figure 1) was removed and a total of 176 qualified CRC samples with clinical data (Supplementary Table 1) were included (Supplementary Figure 2A, the upper panel). Clinical characteristics of tumor pathological stage, histological grade, recurrence, and differentiated status of CRC patients were denoted (Supplementary Figure 2A, the lower panel). After screened by MADs arranged from large to small, the expression of the top 25% genes (5044 genes) with the greatest differences in samples were analyzed by WGCNA.

### WGCNA Construction and Identification of Key Modules for CRC Recurrence

**FIGURE 2:**
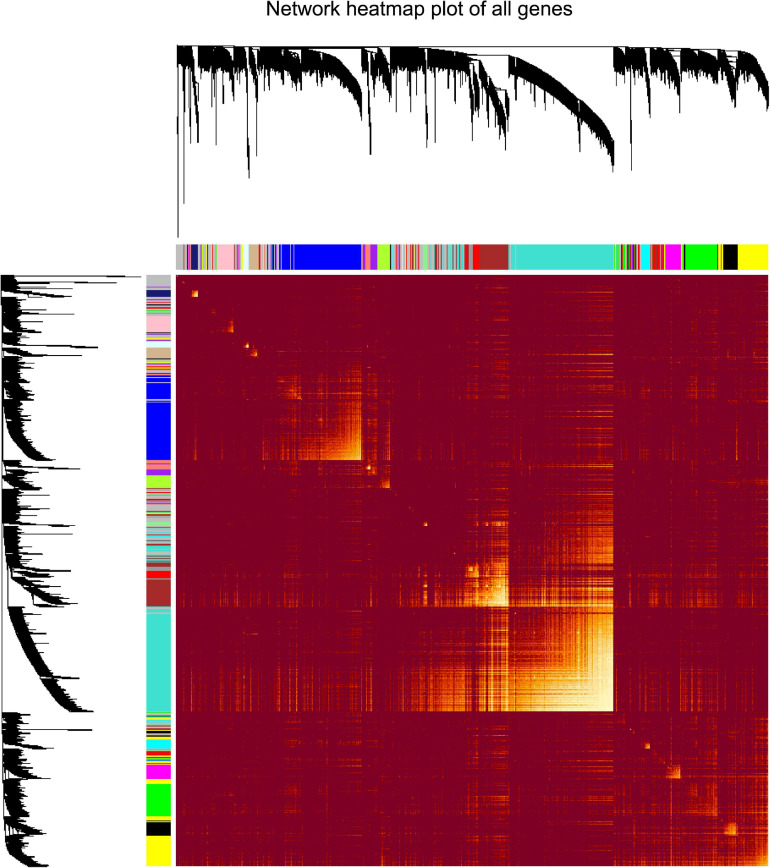
Analysis of co-expressing genes modules. Topological overlap matrix plot of all genes. Genes in the rows and columns are sorted by the clustering tree. Different colors of the horizontal axis and vertical axis represent different modules. The brightness of yellow in the middle represents the degree of connectivity of different modules.

**FIGURE 3:**
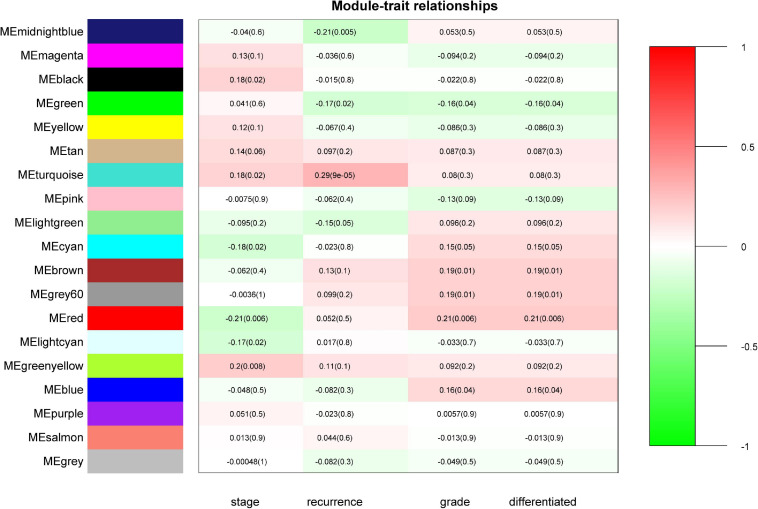
Identification of modules associated with the clinical status of CRC. Heatmap of the correlation between module eigengenes (ME) and clinical traits of CRC. Numbers represent correlation (numbers in brackets are *P*-values). The turquoise module was most positively correlated with CRC recurrence.

“WGCNA” package in R was applied to classify the DEGs with similar expression patterns into modules by average linkage clustering. As a result, a total of 19 modules were identified by merging similar modules when the MedissThres was set at 0.25 (Supplementary Figure 3). The network heatmap is presented in Figure 2. The relevance between the key module and CRC recurrence was tested using two methods. Our results indicated that the ME of the turquoise module possessed the highest correlation with tumor recurrence [(*P* = 9 × 10^–5^, *R*^2^ = 0.29), Figure 3]. Furthermore, we also showed that the MS of the turquoise module was the highest among all modules (Supplementary Figure 4), which was considered to be more associated with tumor recurrence. Therefore, we identified the turquoise module to be a clinically significant module of interest in connection with CRC recurrence in the training set. The correlations between module members and GS in the turquoise module is demonstrated by scatter plots in Supplementary Figure 5.

### GO and KEGG Analyses of the Turquoise Module

**FIGURE 4:**
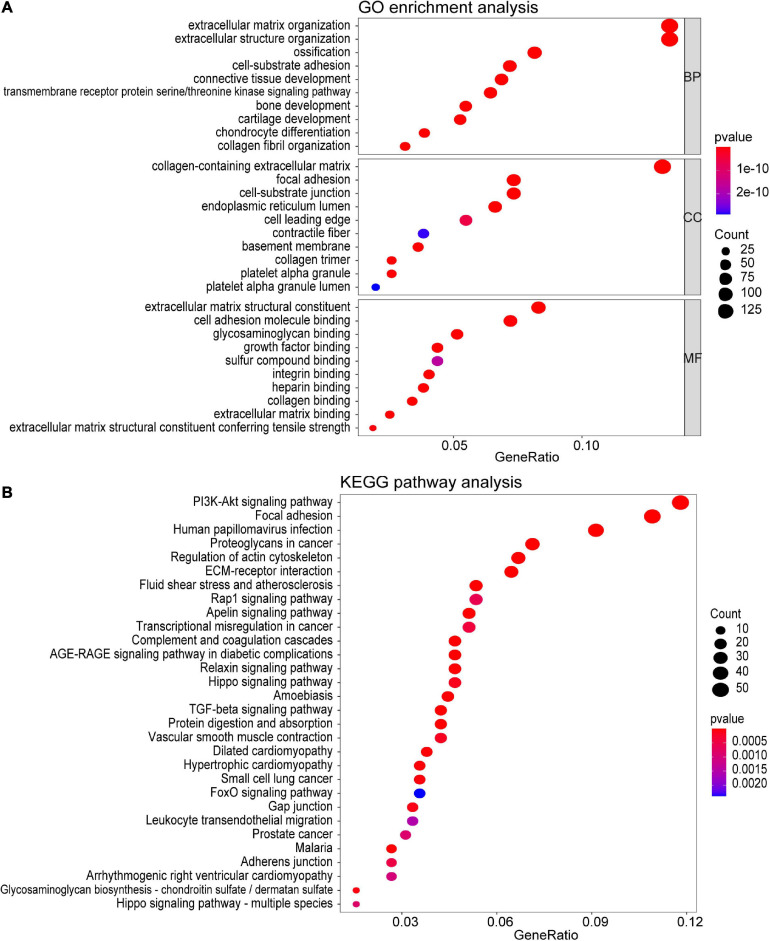
Functional enrichment analysis of all the genes in the turquoise module. **(A)** GO enrichment analysis for biological process, molecular function, and cellular component. **(B)** KEGG pathway enrichment analysis. The colored dots represent term enrichment, blue indicates low enrichment, and red indicates high enrichment. The sizes of the dots represent the number of genes in each GO category and KEGG pathways.

To investigate the potential function of the genes in the turquoise module, GO and KEGG pathway analyses were performed. As shown in Figure 4A, genes in the turquoise module were predicted to exert their functions in the fields of extracellular structure organization, collagen-containing extracellular matrix, and extracellular matrix structural constituent, etc. In addition, these genes were found to be significantly associated with cellular pathways including the PI3K-Akt signaling pathway and regulation of actin cytoskeleton (Figure 4B).

### PPI Network Construction and Key Clusters Analysis of the Turquoise Module

**FIGURE 5:**
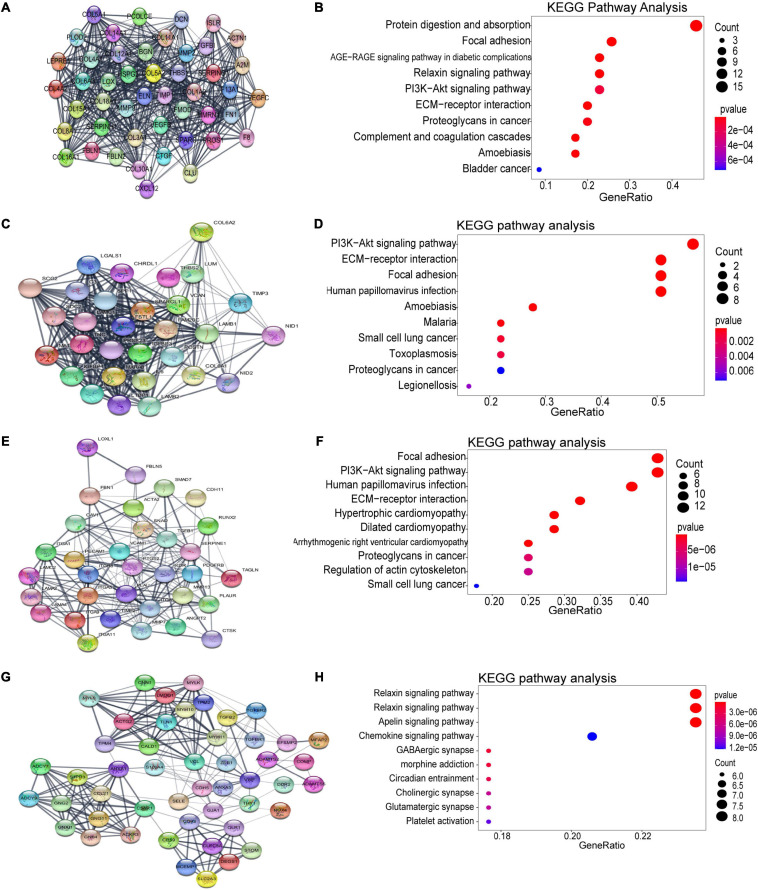
Identification of the top four modules (159 genes in total) and correlated pathways from the PPI network. **(A,B)** Module 1 (47 genes) and its top 10 enriched KEGG pathways. **(C,D)** Module 2 (30 genes) and its top 10 enriched KEGG pathways. **(E,F)** Module 3 (33 genes) and its top 10 enriched KEGG pathways. **(G,H)** Module 4 (49 genes) and its top 10 enriched KEGG pathways.

Using the STRING database and Cytoscape software, a total of 926 DEGs of the turquoise module were mapped into the PPI network, including 926 nodes and 7045 edges. The top four significant clusters within PPI network were selected using MCODE plug-in in Cytoscape software (Clusters 1, MCODE score = 24.435; Clusters 2, MCODE score = 20; Clusters 3, MCODE score = 11.562, Clusters 4, MCODE score = 8.958). The functions of each module were further analyzed (Figure 5). Pathway enrichment analysis demonstrated that Module 1 consisted of 47 nodes and 562 edges (Figures 5A,B), which were mainly associated with protein digestion and absorption, focal adhesion, PI3K-Akt signaling pathway, ECM-receptor interaction, proteoglycans in cancer, and bladder cancer. Module 2 consisted of 30 nodes and 290 edges (Figures 5C,D), which were mainly associated with the PI3K-Akt signaling pathway, ECM-receptor interaction, focal adhesion, and small cell lung cancer. Module 3 consisted of 33 nodes and 185 edges (Figures 5E,F), which were associated with focal adhesion, PI3K-Akt signaling pathway, ECM-receptor interaction, dilated cardiomyopathy, proteoglycans in cancer, regulation of actin cytoskeleton, and small cell lung cancer. Module 4 consisted of 49 nodes and 215 edges (Figures 5G,H), which were associated with the relaxin signaling pathway, vascular smooth muscle contraction, apelin signaling pathway, chemokine signaling pathway, and platelet activation.

### Multiple Identification and Validation of Key Genes With Poor Prognosis for CRC Recurrence

**FIGURE 6:**
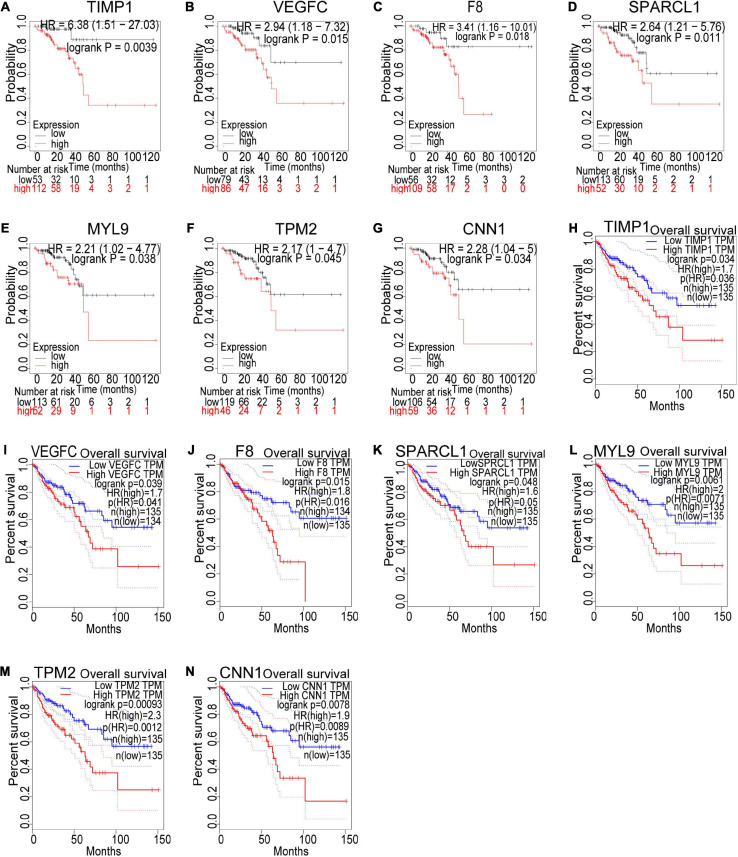
Survival analysis of the seven hub gene candidates identified by the Kaplan–Meier plotter and GEPIA successively. **(A–G)** Overall survival of the seven candidate hub genes in CRC based on Kaplan–Meier plotter. **(H–N)** Overall survival of the seven candidate hub genes in CRC obtained from the GEPIA database. *P* < 0.05 was considered to indicate a statistically significant difference. TIMP1, metallopeptidase inhibitor 1; VEGFC, vascular endothelial growth factor C; F8, coagulation factor VIII; SPARCL1, SPARC Like 1; MYL9, myosin light chain 9; TPM2, tropomyosin 2; CNN1: calponin 1.

**FIGURE 7:**
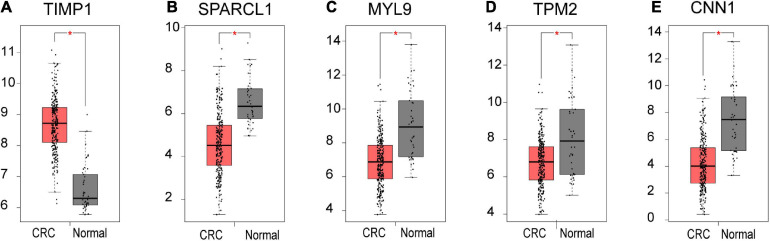
The mRNA level of five hub genes in CRC. Validation of the mRNA expression levels of **(A)** TIMP1, **(B)** SPARCL1, **(C)** MYL9, **(D)** TPM2, and **(E)** CNN1 in CRC tissues compared with normal colon tissues from GEPIA database. These five box plots are based on 275 CRC samples (marked in red) and 41 normal samples (marked in gray). *P < 0.01 was considered statistically significant. CRC, colorectal cancer. VEGFC and F8 were filtered out according to the *P*-value of the mRNA expression levels.

**FIGURE 8:**
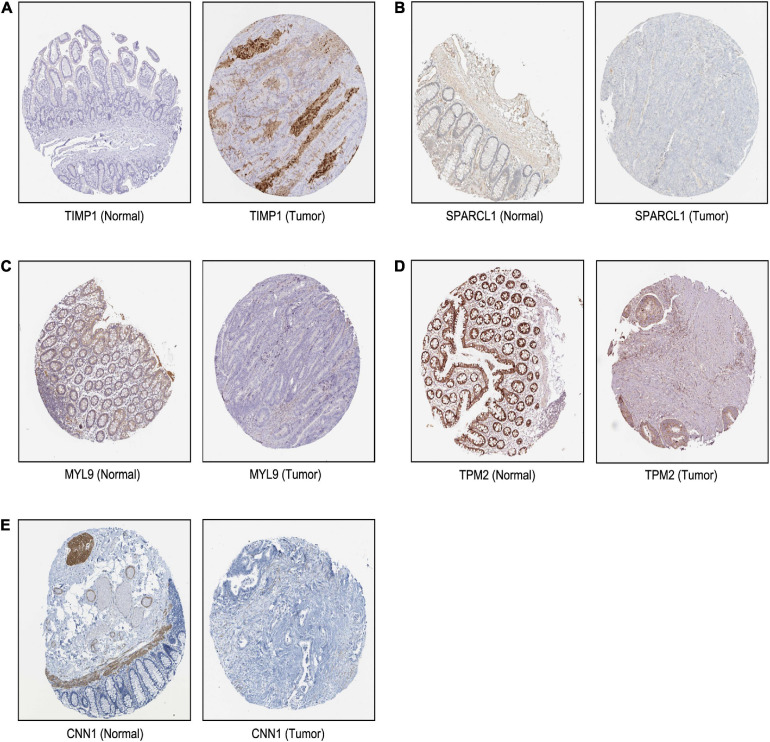
The translational differences of five hub genes in CRC. The expression of **(A)** TIMP1, **(B)** SPARCL1, **(C)** MYL9, **(D)** TPM2, and **(E)** CNN1 on translational levels in CRC tissues compared with normal colon tissues from The Human Protein Atlas database (immunohistochemistry).

Based on our findings above, a total of 159 genes (47 in Cluster 1, 30 in Cluster 2, 33 in Cluster 3, and 49 in Cluster 4, respectively) were identified as potential hub genes. To reduce the scope and further validate these observations, CRC patient survival analysis using Kaplan–Meier plotter was first conducted to unveil the prognostic information of these genes. After the above validation, only genes with significantly deteriorated survival curves were further validated by survival analysis with GEPIA. As shown in Figure 6, the higher expression of seven candidate hub genes was significantly correlated with poor CRC patient survival. Subsequently, in order to further verify the genes identified by the above methods, the expression level of seven specific genes was evaluated by GEPIA. As a result, elevated expression of TIMP1 was observed in CRC samples as compared to normal colon samples, while reduced expressions of SPARCL1, MYL9, TPM2, and CNN1 were found in CRC samples as compared to normal control (Figure 7). Unexpectedly, VEGFC and F8 were filtered out according to the *P*-value of the mRNA expression levels. To further confirm these results, the protein level of these five hub gene candidates was checked using IHC staining data obtained from The Human Protein Atlas database. Consistent with the GEPIA results, IHC data also showed the dysregulated protein level of these genes in CRC tissues (Figure 8). In summary, five candidate genes (TIMP1, SPARCL1, MYL9, TPM2, and CNN1) were finally characterized as the hub gene associated with tumor recurrence in CRC.

**FIGURE 9:**
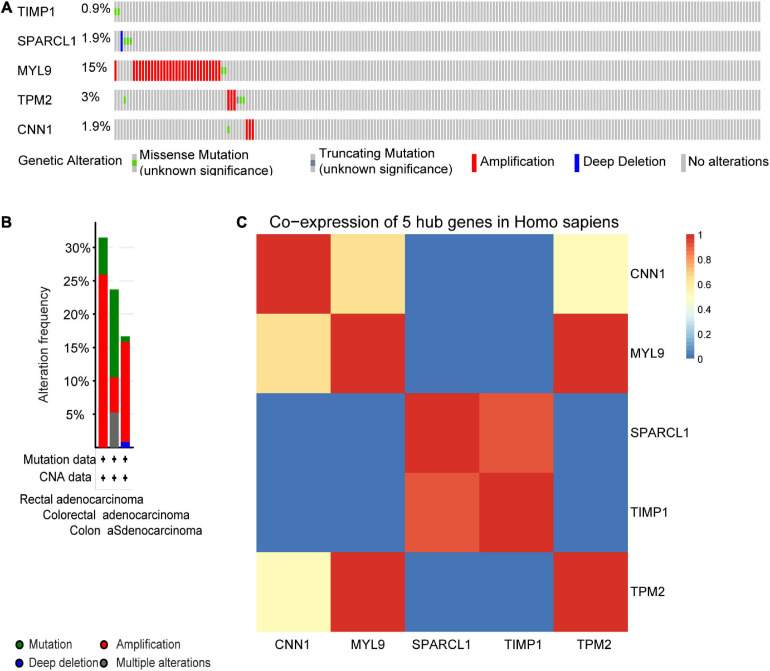
Genetic alteration information and co-expression analysis of the five poor prognostic genes in CRC. **(A)** A visual summary across a set of CRC (data from colorectal adenocarcinoma, TCGA, Nature 2012) showed the genetic alterations connected with the five hub genes which were altered in 49 (22.7%) of 212 sequenced patients (212 in total). **(B)** An overview of changes in the five hub genes in the genomics datasets of CRC in TCGA database. Summary for rectal adenocarcinoma: gene altered in 31.48% of 54 cases, mutation 5.56% (3 in 54 cases), amplification 25.93% (14 in 54 cases). Summary for colorectal adenocarcinoma: gene altered in 23.68% of 38 cases, mutation 13.16% (5 in 38 cases), amplification, 5.26% (2 in 38 cases), multiple alterations5.26% (2 in 38 cases). Summary for colon adenocarcinoma: gene altered in 16.67% of 120 cases, mutation, 0.83% (1 in 120 cases), amplification, 15% (18 in 120 cases), deep deletion 0.83% (1 in 120 cases). **(C)** The co-expression analysis of five hub genes using the STRING online database.

We next sought to compare the genetic alterations of the selected five hub genes in CRC from cBioPortal. As presented in Figure 9A, amplification or missense mutation of the MYL9 gene was observed in 15% of CRC patients, while the genetic alteration level of the other hub genes was relatively low. A combined analysis revealed that genetic alteration of five hub genes was found in over 30% of patients with rectal adenocarcinoma, more than 20% in colorectal adenocarcinoma, and over 15% in COAD (Figure 9B). The gene co-expression analysis of the five hub genes was then performed using STRING database. The result in Figure 9C showed that these genes might be actively interacted with each other, especially for MYL9 and TPM2.

### Construction of TFs-miRNA–Hub Genes Network Associated With CRC Recurrence

**FIGURE 10:**
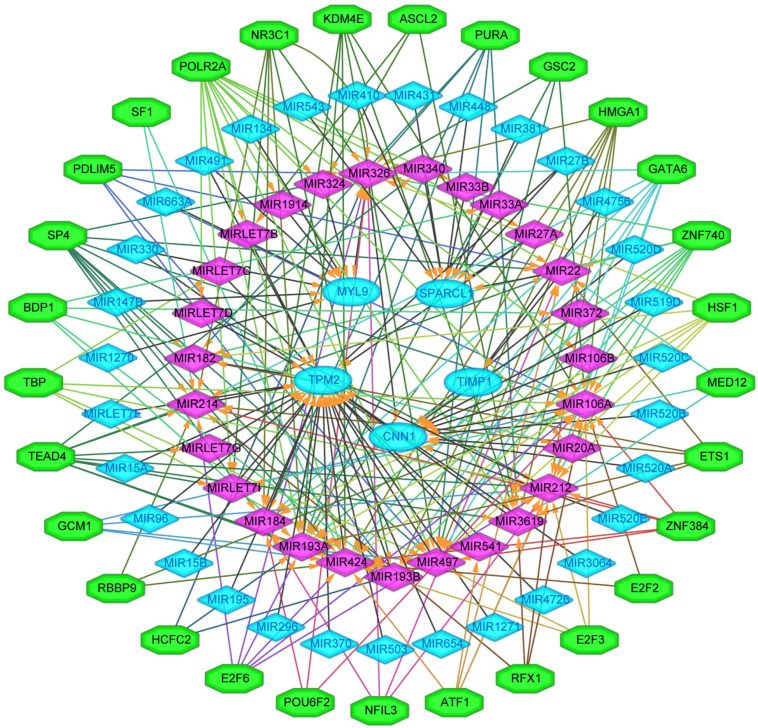
The transcriptional regulatory network of the five hub genes, miRNAs, and TFs. miRNAs, microRNAs; TFs, transcription factors. A green hexagon node represented the TFs, and a pink diamond node represented regulated miRNA, a light blue circular node represented the hub gene, their interaction was represented by an arrow. The numbers of arrows in the networks demonstrate the contribution of one TFs to miRNAs or one miRNA to the hub genes, and the higher the degree the more central the nodes were within the network.

We next sought to establish the transcriptional regulatory network of hub genes, miRNAs, and TFs by starBase. As revealed in Figure 10, a total of 29 TFs, 58 miRNAs, and five hub genes were involved in this network, such as models GATA6-MIR106A-CNN1, SP4-MIR424-TPM2, SP4-MIR326-MYL9, HSF1-MIR424-TPM2, ETS1-MIR22-TIMP1, and ETS1-MIR22-SPARCL1. To further understand the regulatory relationship, central regulatory biomolecules (TFs and miRNAs) were detected using topological parameters (Table 1). A large number of TFs and miRNAs that could regulate hub gene expression may reflect the complexity of the mechanisms that lead to CRC recurrence.

**TABLE 1:** Summary of top 10 regulatory biomolecules (TFs and miRNAs) of the five hub genes in CRC identified from TFs-miRNAs-hub genes interactions.

**Number**	**Symbol**	**Degree**	**Feature**	**References**
	**TFs**			
1	POLR2A	14	Afflicted with cancer	Xu et al., 2019
2	SP4	10	Afflicted with CRC	Meng et al., 2020
3	TEAD4	10	Afflicted with CRC	Kim et al., 2020
4	HMGA1	8	Afflicted with CRC	Yang et al., 2020
5	GATA6	8	Afflicted with CRC	Lai et al., 2020
6	HSF1	7	Afflicted with CRC	Song et al., 2020
7	NR3C1	7	Afflicted with CRC	Sun et al., 2020
8	ATF1	5	Afflicted with CRC	Zhao et al., 2020
9	PDLIM5	5	Afflicted with CRC	Shi et al., 2020
10	ETS1	5	Afflicted with CRC	Xu et al., 2020
11	TBP	5	Afflicted with CRC	Wei et al., 2020
	**miRNAs**			
1	MIR106A	18	Afflicted with CRC	Liu et al., 2020
2	MIR424	17	Afflicted with CRC	Di et al., 2020
3	MIR497	16	Afflicted with CRC	Bai et al., 2020
4	MIR326	16	Afflicted with CRC	Xian et al., 2020
5	MIR212	15	Afflicted with CRC	Mou et al., 2019
6	MIR193A	14	Afflicted with CRC	Hejazi et al., 2020
7	MIR214	11	Afflicted with CRC	Liu et al., 2019
8	MIR22	11	Afflicted with CRC	Cong et al., 2020
9	MIR193B	10	Afflicted with cancer	Tian et al., 2020
10	MIR182	6	Afflicted with CRC	Lin et al., 2020

## Discussion

It is generally accepted that CRC recurrence is possibly the most important factor that influences the survival of CRC patients (Zare-Bandamiri et al., 2017). By performing the effective WGCNA approach, a total of five hub genes were identified to be significantly associated with CRC recurrence in this study. Among them, MYL9 and CNN1 were previously characterized by other groups as CRC recurrence-correlated genes (Qiu et al., 2020), while the others (TIMP1, SPARCL1, and TPM2) have never been reported before.

As a member of the tissue inhibitor of the metalloproteinase (TIMP) family, metallopeptidase inhibitor 1 (TIMP1) was originally discovered as a serum protein that plays a role in collagenase inhibition and erythroid progenitor cell growth (Eckfeld et al., 2019). It has been reported that overexpression of TIMP1 could increase the phosphorylation of the c-Kit and thus promotes proliferation and migration of CRC cells (Nordgaard et al., 2019). Since TIMP1 was expressed mainly in the serum which could be easily detected, its potential role as a CRC diagnostic biomarker has been widely studied (Vocka et al., 2019; Yang et al., 2019). In this study, we further unveiled the association between TIMP1 and CRC recurrence, which may strengthen the clinical relevance of this biomarker in the diagnosis and prognosis of CRC.

Secreted protein acidic and rich in cysteine-like 1 (SPARCL1) belongs to the SPARC-associated family of matricellular proteins and is frequently found to be decreased in a number of cancer types (Gagliardi et al., 2017). Till now, the role of SPARCL1 in CRC is still controversial. Some studies indicated SPARCL1 as a potential CRC suppressor gene that is associated with a good prognosis (Kotti et al., 2014; Han et al., 2018), while others identified SPARCL1 as a potential oncogene in CRC (Zhang et al., 2011). This discrepancy was also observed from our results, in which higher SPARCL1 expression predicts poorer CRC patient survival through Kaplan–Meier analysis, but down-regulated SPARCL1 protein level was observed in CRC tissues using GEPIA and Human Protein Atlas database.

Beta-tropomyosin (β-tropomyosin, TPM2) encodes a thin filament-associated protein which has been proved to play a crucial role in the regulation of muscle contraction (Karpicheva et al., 2016). Single-cell multiomics sequencing revealed TPM2 as one of the fibroblast-specific biomarkers representing a poorer prognosis of CRC (Zhou et al., 2020). Another research suggested that down-regulation of TPM2 was associated with RhoA activation and proliferation of CRC cells (Cui et al., 2016). In the current study, strong interaction was predicted between TPM2 and myosin light chain 9 (MYL9), which deserves to be further investigated to unveil its potential impact on the tumor recurrence of CRC.

In this study, a regulation network of TFs-miRNAs-hub genes was constructed using StarBase and Cytoscape. We first established the regulatory network of TFs-miRNAs-target genes for the recurrence of CRC, involving 29 TFs, 58 miRNAs, and five hub genes, such as models GATA6-MIR106A-CNN1, SP4-MIR424-TPM2, SP4-MIR326-MYL9, HSF1-MIR424-TPM2, ETS1-MIR22-TIMP1, and ETS1-MIR22-SPARCL1. Interestingly, TFs and miRNAs with high connective degrees in regulatory networks have been reported to be closely related to CRC, shown as in Table 1.

## Conclusion

Our current study identified a number of five hub genes including TIMP1, SPARCL1, MYL9, TPM2, and CNN1, which may play vital roles during CRC recurrence. Their PPI network and upstream TF and miRNA regulators were also investigated to unveil the underlying mechanism by which these hub genes modulate the progression of CRC recurrence. It is anticipated that targeting these hub genes solely or combined therapy with currently available anti-cancer drugs may be served as an alternative method to benefit patients diagnosed with CRC. In general, this study may pave a novel way for the diagnosis, prognosis, and treatment strategies of the devastating disease.

## Data Availability Statement

The original contributions presented in the study are included in the article/Supplementary Material, further inquiries can be directed to the corresponding author/s.

## Author Contributions

QD conceived the idea. SL finished the bioinformatics analysis and drafted the manuscript. FZ and GF involved in the data analysis and interpretation. All authors contributed to the article and approved the submitted version.

## Conflict of Interest

The authors declare that the research was conducted in the absence of any commercial or financial relationships that could be construed as a potential conflict of interest.

## Footnotes

1 http://bioconductor.org/packages/release/bioc/html/clusterProfiler.html

2 http://string-db.org/

3 http://cytoscape.org/

4 http://gepia.cancer-pku.cn/

5 http://www.proteinatlas.org

6 http://www.cbioportal.org/

7 http://starbase.sysu.edu.cn/index.php

## Supplementary Material

Supplementary Figure 1 Clustering of samples to detect outliers.

Supplementary Figure 2 Sample clustering tree and soft threshold power analysis in the WGCNA. **(A)** The clustering was based on the expression data in the GSE17536 dataset (*n* = 176). The color intensity was proportional to tumor pathological stage, histological grade, recurrence, and differentiated status. In recurrence status, red means cancer recurrence, white means no recurrence, and gray means unknown. **(B)** Scale independence analysis of the scale-free fit index for various soft-thresholding powers (β). The power of β = 4 was selected. **(C)** Analysis of the mean connectivity for various soft-thresholding powers. determination of soft-thresholding power by analyzing network topology for thresholding powers from 1 to 20. **(D)** Checking the scale free topology when β = 4. K, connectivity; p(k), possibility of the connectivity.

Supplementary Figure 3 Dendrogram of all differentially expressed genes clustered based on a dissimilarity measure (1-TOM). Each branch in the figure represented one gene, and the colors below represented co-expression modules.

Supplementary Figure 4 Module clusters for CRC recurrence. There are total 19 Module memberships vs. gene significance clusters for CRC recurrence.

Supplementary Figure 5 Correlation of the key turquoise module with CRC recurrence. Scatter plot of the correlation between MEturquoise membership and gene significance.
