# Supplementary material for: Identification of Hub Genes and Construction of a Transcriptional Regulatory Network Associated With Tumor Recurrence in Colorectal Cancer by Weighted Gene Co-expression Network Analysis
Source: Front Genet. 2021 Apr 7;12:649752. doi: 10.3389/fgene.2021.649752 (PMC8058478; doi:10.3389/fgene.2021.649752)
Supplement: Supplementary Figure 1 — Clustering of samples to detect outliers. [file Presentation_1.pdf]

Supplemental figure legends

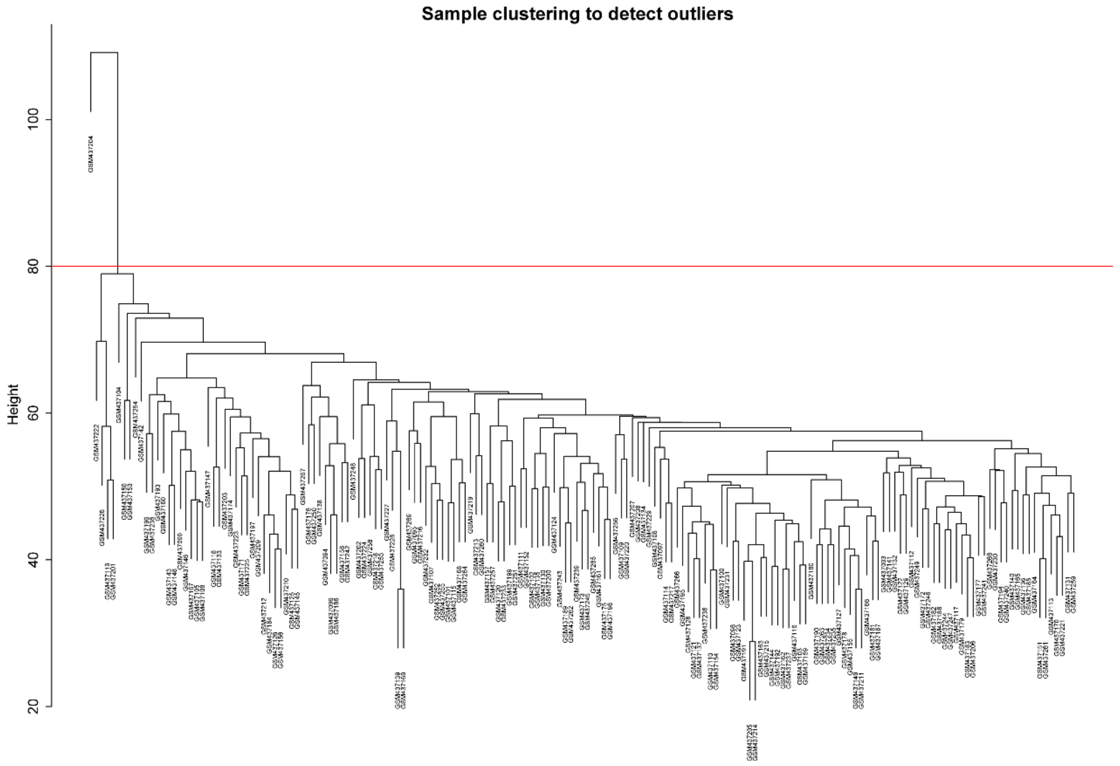

Figure S1 Clustering of samples to detect outliers.

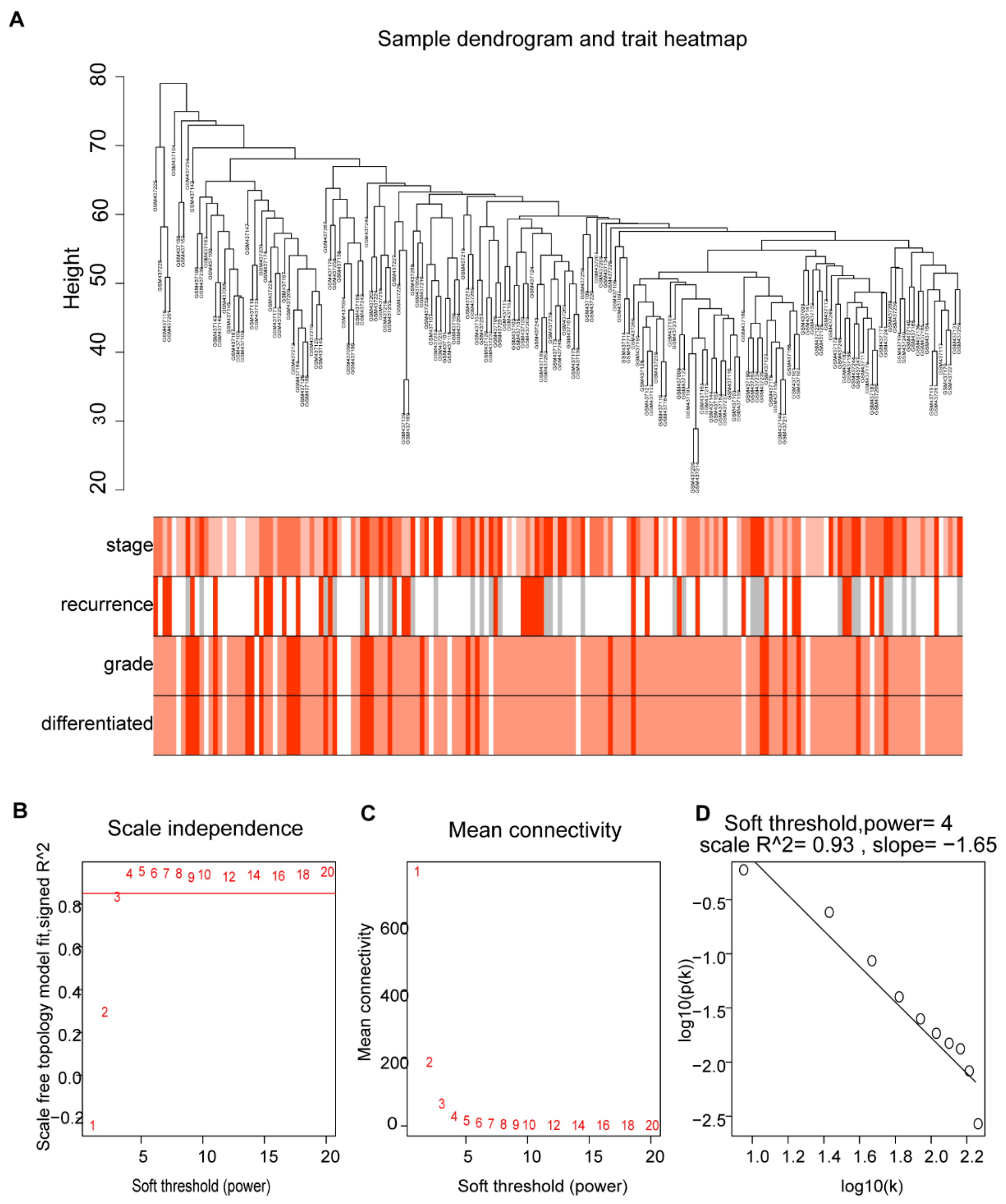

Figure S2 Sample Clustering tree and soft threshold power analysis in the WGCNA.

(A) The clustering was based on the expression data in GSE17536 dataset (n=176). The color intensity was proportional to tumor pathological stage, histological grade, recurrence and differentiated status. In recurrence status, red means cancer recurrence, white means no recurrence and gray means unknown.

(B) Scale independence analysis of the scale-free fit index for various soft-thresholding powers (β). The power of β = 4 was selected.

(C) Analysis of the mean connectivity for various soft-thresholding powers. determination of soft-thresholding power by analyzing network topology for thresholding powers from 1 to 20.

(D) Checking the scale free topology when β = 4. k: Connectivity; p(k): Possibility of the connectivity.

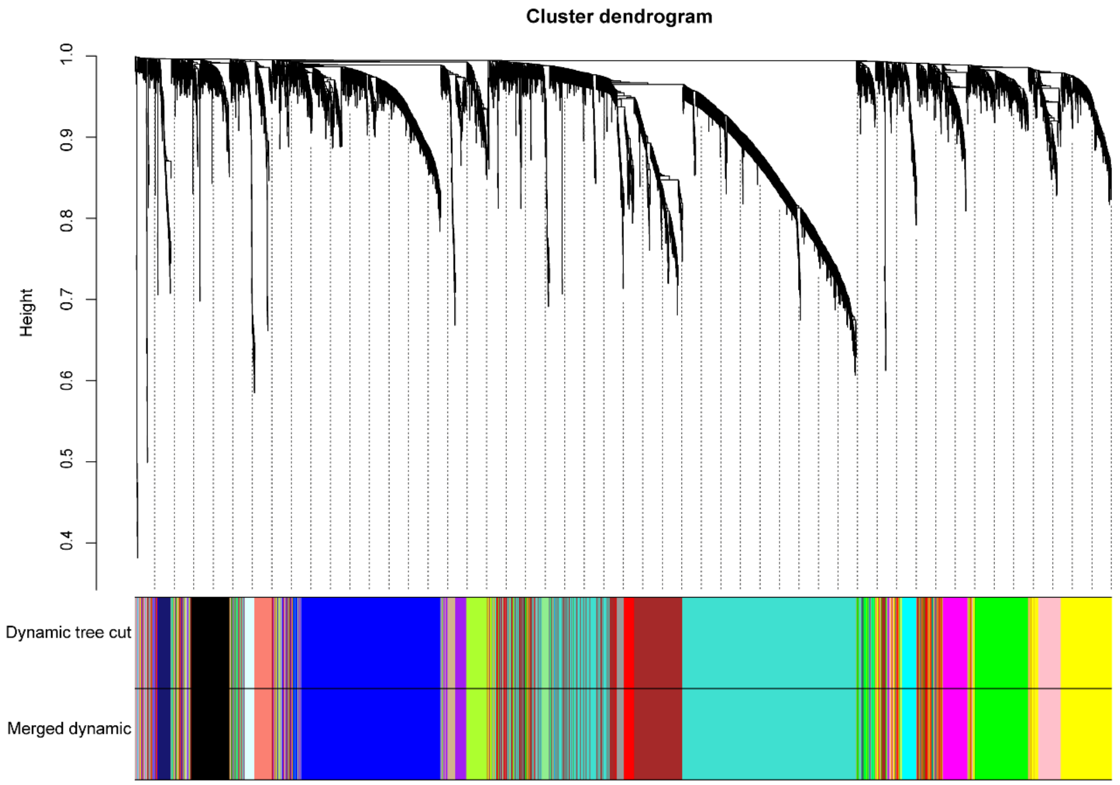

Figure S3 Dendrogram of all differentially expressed genes clustered based on a dissimilarity measure (1-TOM). Each branch in the figure represented one gene, and colors below represented co-expression modules.

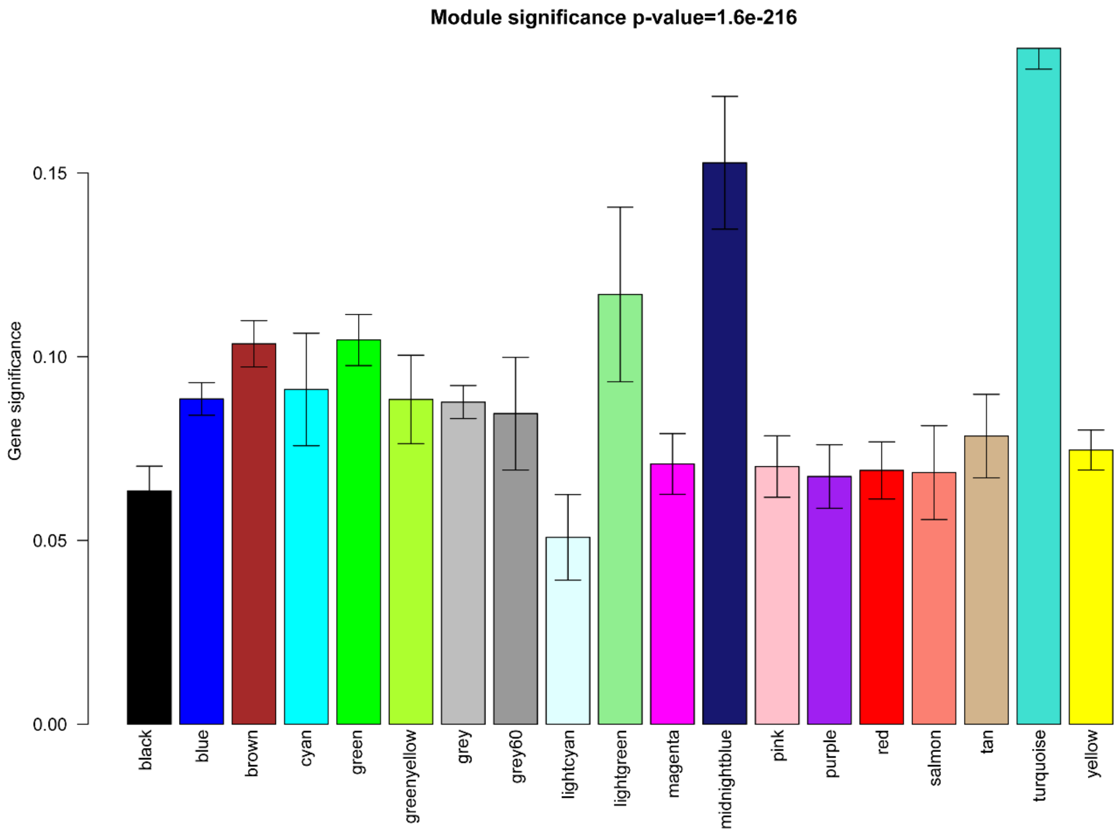

Figure S4 Module clusters for CRC recurrence. There are total 19 Module memberships vs. gene significance clusters for CRC recurrence.

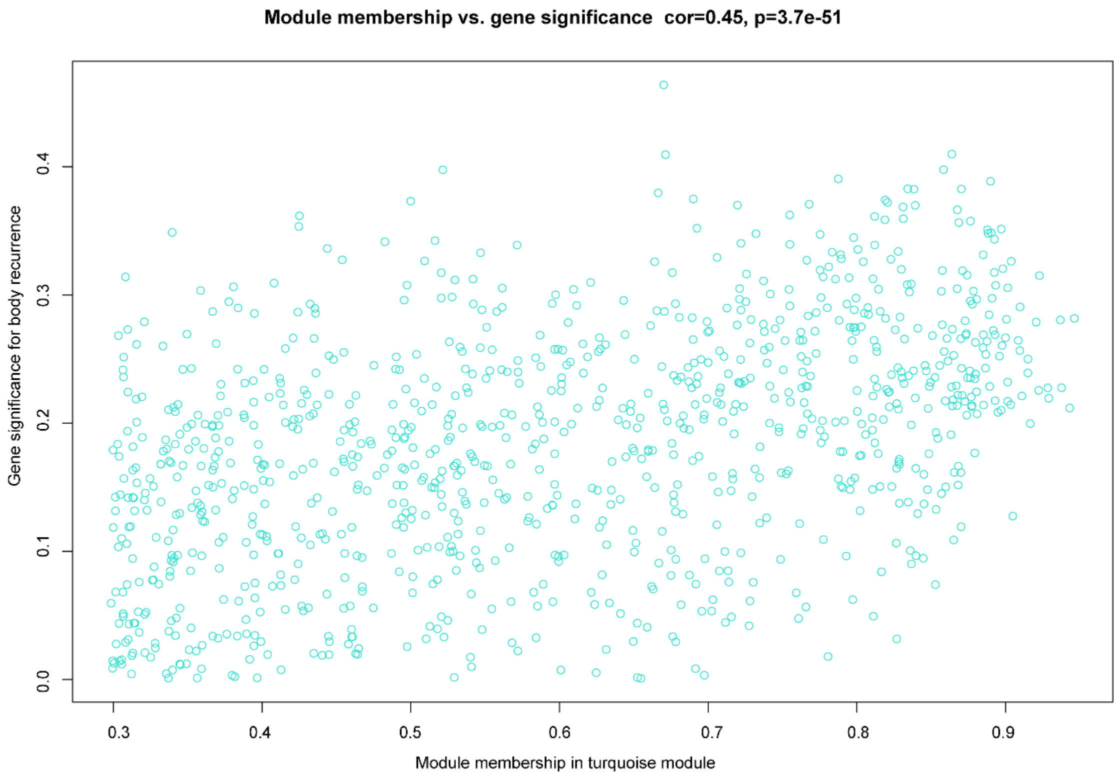

Figure S5 Correlation of the key turquoise module with CRC recurrence. Scatter plot of the correlation between MEturquoise membership and gene signifcance**.**
